# Antibacterial action of penicillin against *Mycobacterium avium complex*

**DOI:** 10.5588/ijtldopen.24.0238

**Published:** 2024-08-01

**Authors:** D. Deshpande, G. Magombedze, S. Srivastava, T. Gumbo

**Affiliations:** ^1^ Baylor University Medical Center, Dallas, TX, USA;; ^2^ Mathematical Modeling and AI Department, Praedicare Inc, Dallas, TX, USA;; ^3^ Department of Medicine, School of Medicine, University of Texas at Tyler, Tyler, TX, USA;; ^4^ Department of Cellular and Molecular Biology, Center for Biomedical Research, University of Texas Health Science Center at Tyler, Tyler, TX, USA;; ^5^ Hollow Fiber System & Experimental Therapeutics Laboratories, Praedicare Inc, Dallas, Texas, USA

**Keywords:** ordinary differential equations, antimicrobial resistance, susceptibility breakpoint, continuous dosing, NTM lung disease

## Abstract

**INTRODUCTION:**

β-lactam antibiotics are promising treatments for *Mycobacterium avium* complex (MAC) lung disease. We hypothesized that benzylpenicillin has efficacy against MAC.

**METHODS:**

Benzylpenicillin lung concentration–time profiles of seven doses in three dosing schedules were administered for 28 days using the hollow fiber system model of intracellular MAC (HFS-MAC). Data were analyzed using the inhibitory sigmoid maximal effect (E_max_) model for each sampling day, while two ordinary differential equations (ODEs) were used for the wild-type and penicillin-resistant mutants.

**RESULTS:**

Benzylpenicillin killed >2.1 log_10_ colony-forming unit (CFU)/mL below Day 0, better than azithromycin, ethambutol, and rifabutin. Efficacy was terminated by acquired resistance. Sigmoid E_max_ parameter estimates significantly differed between sampling days and were a poor fit. However, ODE model parameter estimates vs. exposure were a better fit. The exposure mediating E_max_ was 84.6% (95% CI 76.91–82.98) of time concentration exceeded the minimum inhibitory concentration (MIC). In Monte Carlo experiments, 24 million international units of benzylpenicillin continuous infusion achieved the target exposure in lungs of >90% of 10,000 subjects until an MIC of 64 mg/L, designated the susceptibility breakpoint.

**CONCLUSIONS:**

Benzylpenicillin demonstrated a better bactericidal effect against MAC than guideline-recommended drugs before the development of resistance. Its role in combination therapy with other drugs with better efficacy than guideline-recommended drugs should be explored.

## RÉSUMÉ

**INTRODUCTION:**

Les β-lactamines représentent des options thérapeutiques prometteuses pour le traitement de la maladie pulmonaire à complexe *Mycobacterium avium* (MAC). Notre hypothèse suggère que la benzylpénicilline pourrait être efficace contre cette maladie pulmonaire causée par *M. avium*.

**MÉTHODES:**

Les concentrations pulmonaires de benzylpénicilline ont été mesurées à différents moments après l'administration de sept doses selon trois schémas différents pendant 28 jours dans le modèle de MAC intracellulaire du système de fibres creuses (HFS-MAC). Les données ont été analysées en utilisant un modèle sigmoïde inhibiteur à effet maximal (E_max_) pour chaque jour d'échantillonnage, et deux équations différentielles ordinaires (ODE) ont été appliquées pour les souches sauvages et les mutants résistants à la pénicilline.

**RÉSULTATS:**

La benzylpénicilline a provoqué une réduction de >2,1 log10 CFU/mL en dessous du jour 0, surpassant ainsi l'azithromycine, l'éthambutol et la rifabutine. Cependant, son efficacité a été compromise par l'émergence d'une résistance. Les estimations des paramètres de l'E_max_ sigmoïde ont montré des différences significatives entre les jours d'échantillonnage et étaient mal ajustées. En revanche, les estimations des paramètres du modèle ODE en fonction de l'exposition étaient plus précises. L'exposition médiane de l'E_max_ était de 84,6% (IC à 95% 76,91–82,98) du temps où la concentration dépassait la concentration minimale inhibitrice (MIC, pour l’anglais « minimum inhibitory concentration »). Dans les simulations de Monte Carlo, une perfusion continue de 24 millions d'unités internationales de benzylpénicilline a permis d'atteindre l'exposition cible dans les poumons de plus de 90% des 10 000 sujets, jusqu'à ce qu'une MIC de 64 mg/L soit atteinte, indiquant ainsi le point de rupture de la sensibilité.

**CONCLUSIONS:**

La benzylpénicilline a montré une efficacité bactéricide supérieure contre le MAC par rapport aux médicaments recommandés par les lignes directrices avant l'émergence de la résistance. Il convient d'explorer son utilisation dans une thérapie combinée avec des médicaments plus performants que ceux recommandés par les lignes directrices.

Soon after the discovery of penicillin, Alexander Fleming and others attempted to use it for mycobacteria in the 1940s, specifically *Mycobacterium tuberculosis* (MTB) and found the drug to have no effect.^1–3^ In 2017, we demonstrated that >64 mg/L of static benzylpenicillin concentrations killed >1.0 log_10_ colony-forming unit (CFU)/mL of MTB below Day 0 (stasis) in combination with the β-lactamase inhibitor (BLI) avibactam.^4^ However, recent work with ceftriaxone and ertapenem demonstrated no advantage to adding avibactam, likely due to the lack of MTB’s BlaC homolog in *Mycobacterium avium* complex (MAC).^5,6^ Here, we hypothesized that benzylpenicillin could be effective against MAC without the need for avibactam.

MAC lung disease (MAC-LD) is difficult to treat with the standard-of-care (SOC) macrolide, ethambutol (EMB), and rifamycin combination regimen. Intention-to-treat meta-analyses have demonstrated that macrolide-based therapy achieves sustained sputum culture conversion (SSCC) rates of only 43–53% at 6 months.^7,8^ Moreover, SOC is associated with significant adverse events in 70% of patients, leading to treatment discontinuation in 30%.^9^ To identify more efficacious therapies, we have developed a hollow fiber system model of MAC-LD (HFS-MAC)-based multistep program and validated it using a wide range of antibiotics.^10–15^ The HFS-MAC mirrors the essential pharmacokinetics (PK)/pharmacodynamic (PD) requirements of intracellular MAC in monocyte-lineage cells in lung lesions, and the median initial bacterial burden (B_0_) of 5.18 log_10_ CFU/mL (range: 4.23–6.20) in lung cavitary lesions.^16,17^ However, the program has not been as informative in telling us how the MAC response in terms of CFU/mL/day in the HFS-MAC translates to bacterial response dynamics in patients. Inspired by our modelling and simulation work with MTB, in this study, we used a quantitative translation pathway from the HFS-MAC to patients in terms of predicted time to SSCC in the clinic based on two sets of ordinary differential equations (ODEs) for benzylpenicillin HFS-MAC data.^5,10,18,19^

## METHODS

Materials and bacterial strains, broth micro-dilution MIC^20^ and static concentration-response experiments are described in detail in the Supplementary Data.

The intracellular HFS-MAC model has been described in detail in the past.^11,12,15^ Benzylpenicillin was administered using three dose schedules: the lowest dose (R_2_) was administered once daily, while R_3_–R_7_ were administered twice daily, and R_8_ was administered continuously. The target exposures, measured as the percentage of time the concentration remained above the MIC (%T_MIC_), were as follows: R_1_ at 0%, R_2_ at 20%, R_3_ at 40%, R_4_ at 50%, R_5_ at 60%, R_6_ at 66%, R_7_ at 75%, and R_8_ at 100%. Both central and peripheral compartments were sampled to measure steady-state penicillin concentrations on Day 28, at 1, 2, 3, 5, 12, 13, 19, 21, and 23.5 h post-drug infusion. Benzylpenicillin concentrations were measured using previously reported assays.^21^ The peripheral compartment was sampled on Days 0, 2, 7, 14, 21, and 28 to estimate the bacterial burden on Middlebrook 7H10 agar. In order to capture the emergence of drug resistance, we also cultured the samples on Middlebrook 7H10 agar supplemented with 6 mg/L benzylpenicillin (i.e., three times MIC).

The ODEs are described in detail in the Supplementary Data. Data generated in the current study were analyzed using these ODEs. Data from previously published HFS-MAC studies with azithromycin (AZM), EMB, rifabutin, and AZM–EMB combination^13–15,^^22^ were re-analyzed using the new ODEs. Monte Carlo experiments (MCE) for dose-finding were performed as described in detail in the past and in the Supplementary Data.^5,15,23–25^

The relationship between MAC burden on each sampling day and benzylpenicillin concentrations or exposure (%T_MIC_) was examined using the inhibitory sigmoid E_max_ model:^10^

$$\text{Effect}\left(\log_{10\;}\text{CFU/mL}\right)=\left[{\text{E}}_{\text{con}}-{\text{E}}_{\max}*{\text{EC}}^{H}\right]/\left[{\text{EC}}^{H}+{\text{EC}}_{50}^{\text{H}}\right]$$ [1]

where E_con_ is bacterial burden in non-treated controls, E_max_ is maximal effect, EC_50_ is exposure mediating 50% of E_max_, and H is the Hill factor. In further modelling, we replaced MAC log_10_ CFU/mL with ODE-derived kill-slopes (*γ*). The exposure mediating 80% of E_max_ (EC_80_), which is the target exposure to be achieved in the lung, was derived from *γ*-based inhibitory sigmoid E_max_ models.

### Ethical approval

Ethics approval was not required as the entire study was conducted in in vitro and in *in silico* patients.

## RESULTS

The benzylpenicillin MICs for the MAC American Type Culture Collection strain were 2 mg/L with and without avibactam. Benzylpenicillin MIC was 0.5 mg/L for one and 2 mg/L for four clinical MAC isolates. Supplementary Figure S1 shows concentration–effect results in 24-well plates. Consistent with time-driven activity, E_max_ was achieved between two and four times the MIC. We tested the null hypothesis that E_max_ and EC_50_ were similar with and without avibactam. The E_max_ was statistically similar at 1.32 log_10_ CFU/mL vs. 1.31 log_10_ CFU/mL (*P* = 0.98), while the EC_50_ was 0.06x MIC for benzylpenicillin alone and 4.13x MIC when combined with avibactam (*P* = 0.02). The null hypothesis was rejected because of a better EC_50_ without avibactam compared to with. Therefore, in subsequent HFS-MAC studies, benzylpenicillin was administered without avibactam.

Supplementary Figure S2 shows the drug concentration measurements in the central compartment of each HFS-MAC unit. PK parameters were best described by a two-compartment model (extra- and intra-cellular) with an extracellular clearance of 0.07 ± 0.02 L/h and a volume of 0.31 ± 0.09 L, translating to a half-life of 2.98 ± 1.28 h. The intracellular compartment clearance was 0.002 ± 0.001 L/h, and volume was 0.004 ± 0.003L, translating to a half-life of 1.88 ± 0.06 h. The median ratio of intracellular vs. extracellular penicillin concentration at each time point was 29.43 (95% CI 22.93–64.68). Further details are provided in Supplementary Figure S2.

**Figure 1.**
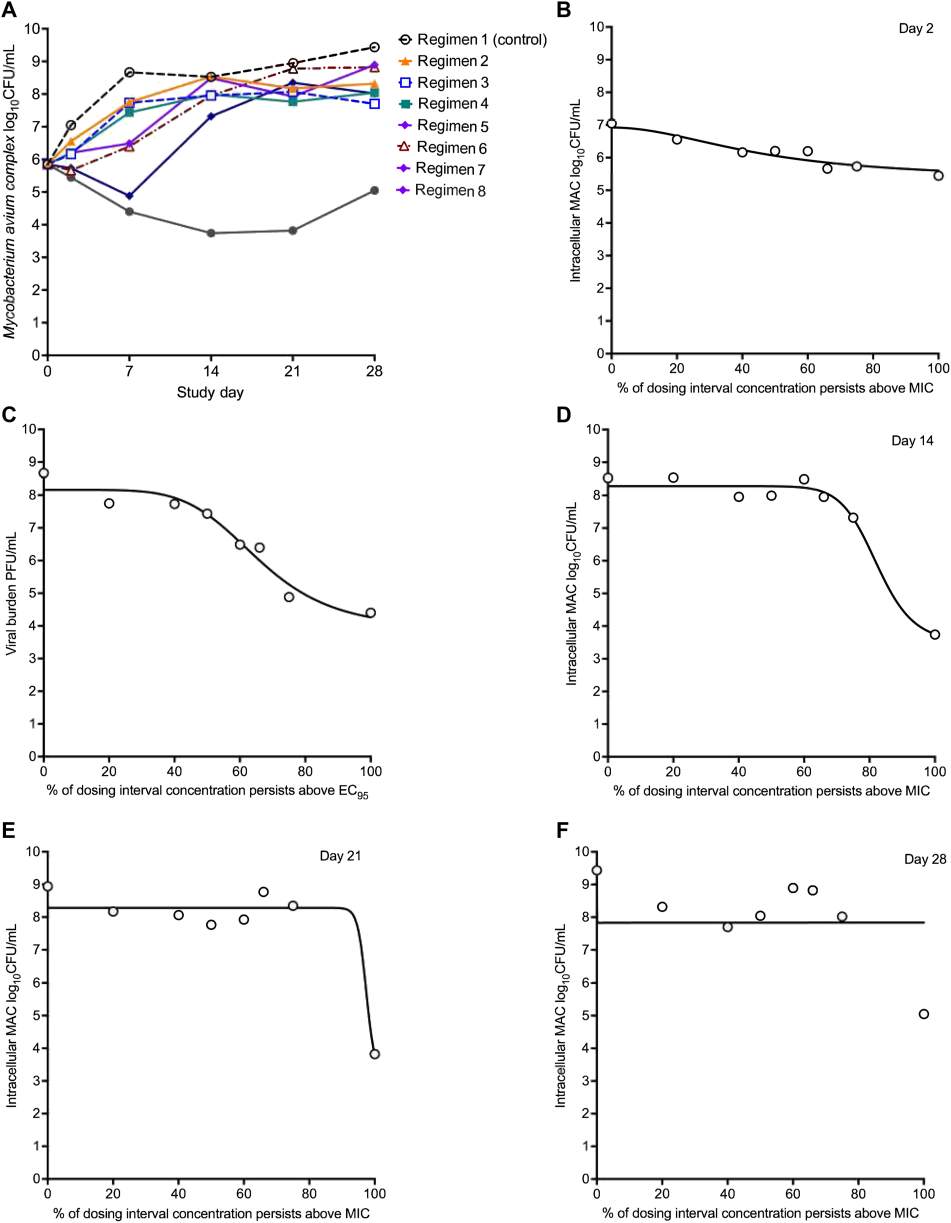
PK/PD effects of benzylpenicillin in the HFS-MAC using traditional approaches. **A)** The benzylpenicillin regimens resulted in the following percentages of time during which the concentration remained above the MIC (%T_MIC_): Regimen 1, 0%; Regimen 2, 20%; Regimen 3, 40%; Regimen 4, 50%; Regimen 5, 60%; Regimen 6, 66%; Regimen 7, 75%; and Regimen 8, 100%. Time-kill curves in HFS-MAC units show that the three top exposures with %T_MIC_ >60% killed MAC below Day 0 (stasis), but then progressively failed. **B)** Inhibitory sigmoid E_max_ models for Day 2, **C)** Day 7, **D)** Day 14, and **E)** Day 21 demonstrated a shifting EC_50_ and varying Hill slopes. **F)** On Day 28, there was no model convergence. MIC = minimum inhibitory concentration; CFU = colony-forming unit; MAC = *Mycobacterium avium* complex; PK/PD = pharmacokinetics/pharmacodynamics; HFS-MAC = hollow fiber system model of intracellular MAC; E_max_ = maximal effect; EC_50_ = exposure mediating 50% of E_max_.

Time-kill curves in the HFS-MAC are shown in Figure 1A. The Day 0 bacterial burden (B_0_) was 5.85 log_10_ CFU/mL, in the range encountered in human cavitary MAC.^16,17^ Benzylpenicillin killed >2.1 log10 CFU/mL below B_0_. For comparison, results for AZM, EMB, and rifamycins from prior HFS-MAC work are shown in Supplementary Figure S3, with a biphasic response for AZM (maximal kill 0.48 log_10_ CFU/mL below B_0_), while EMB and rifabutin did not kill below B_0_.

Inhibitory sigmoid E_max_ model results are shown in Supplementary Table S1. Overall, the inhibitory sigmoid E_max_ models for each sampling day demonstrated poor model fit to the data. Therefore, we constrained the E_max_ to be equal to that observed on each sampling day, which resulted in better fit and inhibitory sigmoid E_max_ curves for several sampling days (but not all) shown in Figure 1B–F. Supplementary Table S2 shows that while there was an improvement in model fit, model parameter estimates changed from one sampling day to another, with poor model convergence for Days 21 and 28 (Figure 1E–F).

The H estimates ranged from 1.99 to 73.37, falling outside the 95% confidence intervals, raising concerns about which sampling day should be used to calculate optimal exposure. The proportion of benzylpenicillin-resistant subpopulation to the total MAC burden in the inoculum was 0.068. The Supplementary Figure S4A shows benzylpenicillin-resistant CFU/mL results that are complete for only non-treated and R_8_ (highest dose) because we did not do enough ten-fold dilutions for R_2_–R_6_ series, so even our most diluted samples had too numerous CFUs to count. This allowed us to calculate drug-resistant populations only as limits, >5.0 log10 CFU/mL on Day 7 and >6.0 log_10_ CFU/mL on Days 14, 21, and 28. Supplementary Figure S4B shows that even in HFS-MAC units treated with the highest dose (R_8_), the total bacterial population was replaced by a penicillin-resistant population by Day 21. Supplementary Figure S4C shows Day 2 results vs. exposure (the only day we had complete resistance data for all regimens) using the antibiotic resistance arrow-of-time model.^15^ The *r*^2^ was 0.80 for all data and 0.94, with one outlier automatically eliminated by the regression program. This means that no tested benzylpenicillin exposure could suppress acquired antimicrobial resistance (AMR).

**Figure 2.**
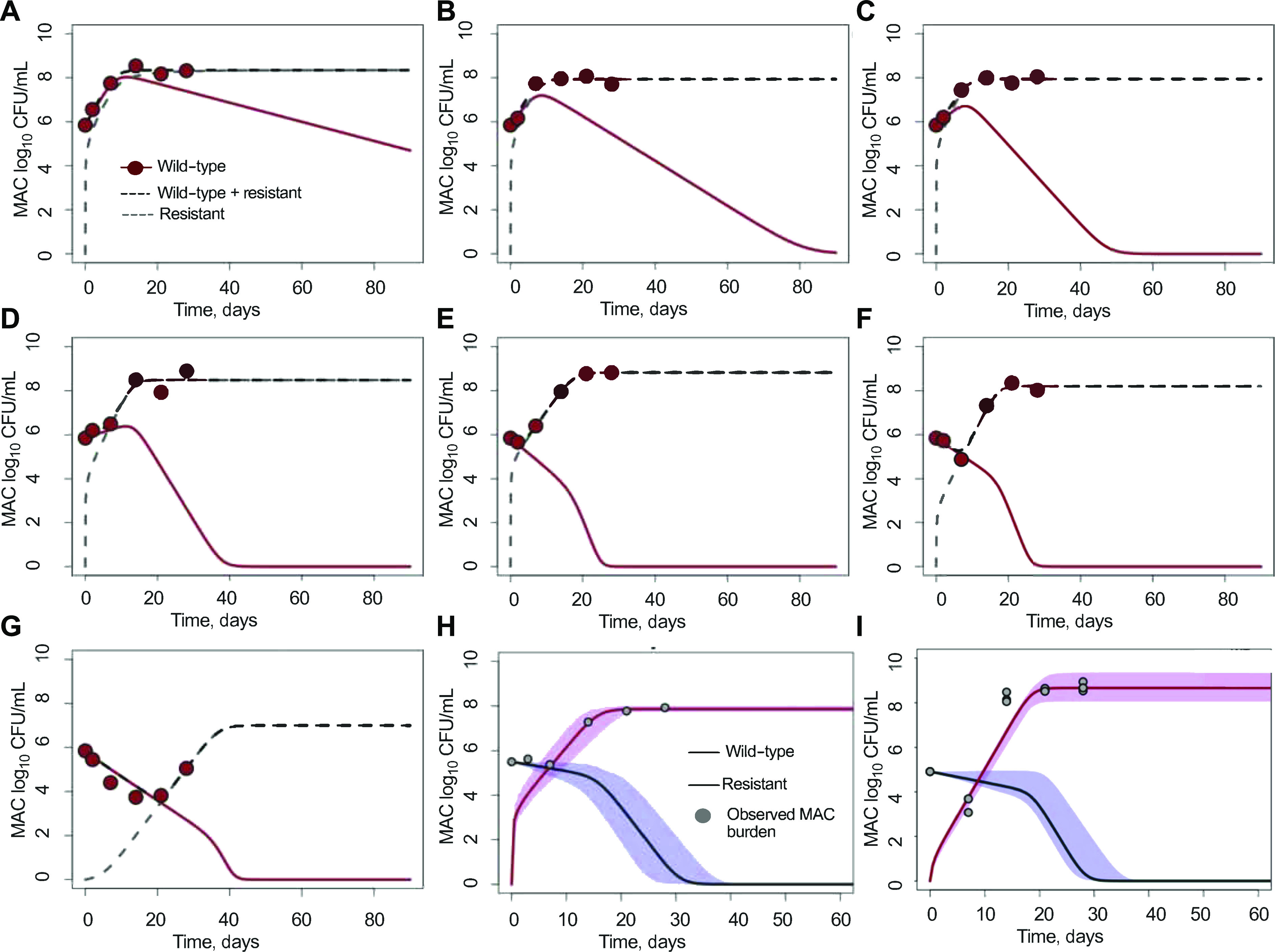
Trajectories of total bacterial burden and drug-resistant MAC based on ODEs. The benzylpenicillin exposures for each regimen expressed as percentages of time during which the concentration remained above MIC (%T_MIC_) as follows: **A)** Regimen 2, 20%; **B)** Regimen 3, 40%; **C)** Regimen 4, 50%; **D)** Regimen 5, 60%; E) Regimen 6, 66%; **F)** Regimen 7, 75%; and **G)** Regimen 8, 100%, vs. bacterial burden (total burden - black dashed) and benzylpenicillin-resistant (grey dashed) vs. wild type (cayenne solid) which is also drug-susceptible. Figure 2 shows that data can be explained assuming mutation and increased kill with increasing drug exposures. As penicillin concentration exposure increased, the size of the drug-resistant subpopulation was reduced. **H** and **I)** The black solid line with the periwinkle shaded area as its 95% credible intervals is the wild-type, while the pink and its 95% credible intervals are the drug-resistant or mutant (drug-resistant) population. **H)** Azithromycin monotherapy at exposures similar to those achieved in humans treated with 500 mg/day is shown for three replicates, showing that the effect was upended by the emergence of azithromycin resistance. **I)** Azithromycin (500 mg/day) plus ethambutol (15 mg/kg/day) combination is shown for three replicates. The data presented indicate ethambutol resistance, demonstrating that the efficacy of the dual regimen was also compromised by this resistance. MAC = *Mycobacterium avium* complex; CFU = colony-forming unit; MIC = minimum inhibitory concentration; ODE = ordinary differential equation.

Figure 2 and the Table show the ODE-based trajectories and parameters of total bacterial burden and the benzylpenicillin-resistant subpopulation. In Figures 2A–G, because of low penicillin exposures achieved in R_2_ to R_5_ (%T_MIC_ 20–60%), trajectories demonstrate only constraining of growth rates and failure to kill below B_0_. As exposures increased in R_6_ to R_8_ (%T_MIC_ 66–100%), the trajectories fell below B_0_ because of the ability to suppress AMR subpopulation. The R_6_ to R_8_ trajectories demonstrated biphasic responses, a different direction for Days 2, 7, and 14, followed by a change in direction due to the emergence of exposure-dependent AMR. In R_8_ (γ = 0.85 log_10_ CFU/mL/day), while the overall trajectory was still in the direction of microbial kill, there would be a failure to achieve bacterial population extinction, and time-to-extinction would be at infinity because of AMR. This means that SSCC cannot be achieved by benzylpenicillin monotherapy. Next, we applied the ODEs to previously published data on AZM monotherapy and AZM–EMB combination (Figure 2H–I).^14^ Supplementary Table S3 shows that AZM monotherapy γ was lower and mutation *m* was higher than for benzylpenicillin R_6_ and R_7_ exposures in the Table and that adding EMB improved γ by reducing m to levels similar to R_6_.

**Table.** Parameter estimates (95% confidence interval) for ordinary differential equations in HFS-MAC regimens.

Parameter	Regimen 1 (95% CI)	Regimen 2 (95% CI)	Regimen 3 (95% CI)	Regimen 4 (95% CI)	Regimen 5 (95% CI)	Regimen 6 (95% CI)	Regimen 7 (95% CI)
*r*_w_	0.75	Fixed	Fixed	Fixed	Fixed	Fixed	Fixed
*r*_m_	0.76 (0.70–0.79)	0.77 (0.69–0.79)	0.75 (0.64–0.79)	0.74 (0.58–0.79)	0.54 (0.52–0.56)	0.67 (0.51–0.74)	0.25 (0.18–0.41)
*m*	0.36 (0.30–0.50)	0.44 (0.33–0.49)	0.124 (0.08–0.15)	0.008 (0.005–0.01)	0.0064 (0.005–0.075)	1.6e–3 (4e–4–9e–3)	4.4e–5 (1e–6–7.4e–5)
*K*_max_	8.34 (8.20–8.47)	7.96 (7.81–8.05)	7.94 (7.85–8.02)	8.49 (8.15–8.85)	8.82 (8.60–8.85)	8.20 (7.88–8.51)	8.13 (7.2–9.0)
γ_s_	0.003 (0.001–0.01)	0.35 (0.26–0.49)	0.55 (0.40–0.79)	0.56 (0.41–0.77)	0.99 (0.97–0.99)	0.97 (0.89–0.99)	0.98 (0.95–0.99)

HFS-MAC = hollow fiber system model of intracellular *Mycobacterium avium* complex; CI = confidence interval; *r*_w_ = growth rate for wild type; *r*_m_ = growth rate for mutant; *m* = mutant strain seeding rate; *K*_max_ = growth limiting capacity; γ_s_ = bacterial kill rate log_10_ CFU/mL/day.

**Figure 3.**
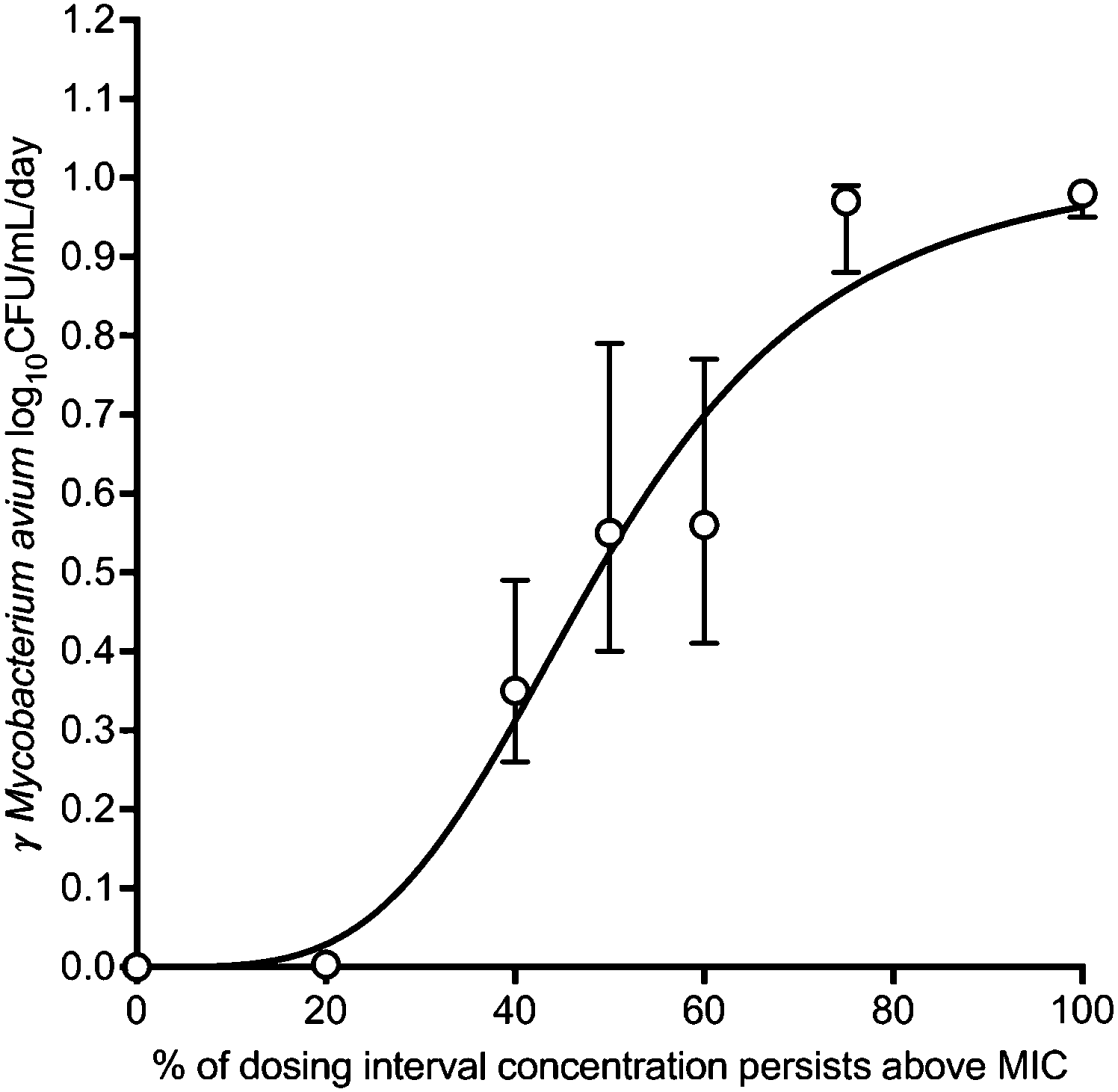
Exposure-response using γ as a pharmacodynamic response parameter. γ is shown on *y*-axis, while benzylpenicillin exposure is shown on the *x*-axis. Error bars indicate 95% confidence intervals, and symbols are mean estimates. The inhibitory sigmoid E_max_ model regression was robust and yielded parameter estimates with narrow confidence intervals and an *r*^2^ = 0.97; there was automatic outlier elimination of one data point by regression programme. CFU = colony-forming unit; MIC = minimum inhibitory concentration.

Next, we used γ as the PD response parameter vs. exposure in the inhibitory sigmoid E_max_ model: a single equation for all data points.^19^ The best Akaike Information Criterion scores (AIC) for PK/PD drivers was %T_MIC_ (−38.79). Results for %T_MIC_ vs. γ are shown in Figure 3. The parameter estimates were an γ E_max_ of 1.02 (95% confidence interval [CI] 0.82–1.22) log_10_ CFU/mL/day, EC_50_ %T_MIC_ of 59.40 (95% CI 54.0–58.26), H of 3.92 (95% CI 1.03–6.82), with an γ E_con_ of 0 (*r*^2^ = 0.97). The γ-based EC_80_ was calculated as %T_MIC_ of 84.6% (95% CI 76.91–82.98).

**Figure 4.**
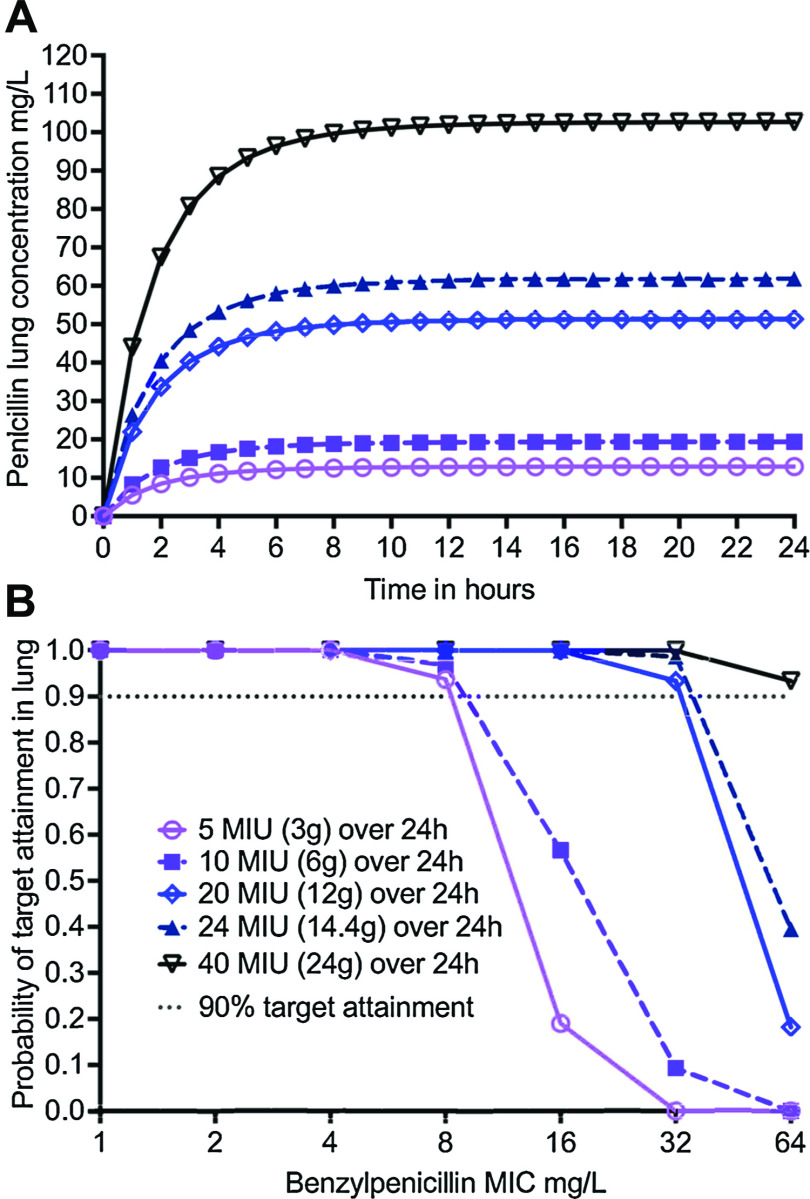
Probability of target attainment for different benzylpenicillin doses. **A)** Lung concentrations are shown as mean concentrations; the 95% confidence intervals are very narrow and smaller than the symbol size. The different doses are embedded in panel B. **B)** PTA at different penicillin MICs. A dose of 24 MIU (14.4 g) achieves PTA of 90% or more at MICs up to 32 mg/L, but for MICs >32 mg/L, a higher dose of 40 MIU (24 g) is needed. MIU = million international units; MAC = *Mycobacterium avium* complex; PTA = probability of target attainment.

The γ-derived EC_80_ %T_MIC_ of 84.6% was used as a target exposure in MCE to identify the probability of target attainment (PTA) in the lungs of 10,000 virtual patients treated with benzylpenicillin doses of 5, 10, 20, and 24 million international units (MIU)/day administered as a continuous infusion. Figure 4A shows the early achievement of steady-state concentrations in the lungs of 10,000 virtual subjects. Figure 4B shows a good PTA at the MICs of 1–4 mg/L. For a dose of 5–10 MIU/day (3–6 g/day), the PK/PD susceptibility breakpoint was calculated as 8 mg/L, and the susceptibility breakpoint was 32 mg/L for 20–24 MIU/day (12–14.4 g/day) and 64 mg/L for 40 MIU/day (24 g/day).

## DISCUSSION

First, when an organism is susceptible to a β-lactam, the effect is usually microbial kill instead of just slowing bacterial growth. The advantage of penicillin is that the drug has low rates of adverse events.^15^ The benzyl ester of penicillin achieves 2x higher lung concentrations than serum.^23,24^ These PK properties and the HFS-MAC PK/PD studies reported here demonstrate >2.0 log_10_ CFU/mL kill as monotherapy. This suggests that benzylpenicillin could be important for rapidly killing intracellular MAC in lung disease in combination therapy. We found that avibactam did not improve the benzylpenicillin anti-MAC effect, consistent with ceftriaxone and ertapenem studies.^4–6,26^ The welcome practical effect is that there will be no need to co-administer a β-lactamase inhibitor in the case of MAC. However, given the intravenous administration of benzylpenicillin, efforts should be made to develop a more user-friendly formulation such as oral administration.

Second, the development of PK/PD indices based on each sampling day for microbes has been the bedrock of PK/PD science.^27^ Such PK/PD concepts were developed with rapidly growing organisms, where doubling time (usually 20 min) was often shorter than the half-life of the drug being tested. This produced highly consistent inhibitory sigmoid E_max_ parameter estimates and a single PK/PD driver linked to the effect that lasted only a few days. In MAC, with a doubling time of ∼16.3 h, and in other slow-growing mycobacteria, the PK/PD parameter linked to efficacy and the inhibitory sigmoid E_max_ parameter estimates demonstrated a change from sampling day to day outside the 95% CIs.^5,19,28^ Our approach using γ is integrative of all time and data points, incorporates the bacterial physiology and the drug-resistant subpopulation, and is also mechanistic, leading to a single equation of exposure versus effect (γ) that yielded narrow 95% CIs and an *r*^2^ > 0.99. We propose γ as the main PD response parameter that could have greater utility in MAC than the traditional approaches.

Third, the mathematical model (ODEs) with wild-type and AMR MAC subpopulations better explained the observed MAC treatment dynamics. The proportion of benzylpenicillin-resistant subpopulation at baseline in the HFS-MAC was 0.068 (i.e., 6.8 × 10^–2^), which may look high and perhaps artefactual compared to proportions identified in the more rapidly growing bacteria and MTB that are in the range of 10^–7^.^4^ However, MAC is known to have very high mutation frequencies, greater than 50% have resistance to rifampin (a first line MAC drug) at proportion of 0.01. Thus, the emergence of AMR to benzylpenicillin monotherapy in the HFS-MAC is not surprising. The model predicts rapid development of benzylpenicillin resistance at low exposures. As drug exposures increased, there was a delayed emergence of AMR. However, the high kill rates alone did not eliminate the AMR subpopulation, even at high penicillin exposures. Model fitting to data suggests that benzylpenicillin mechanism of action could be 1) direct killing of MAC, 2) reducing MAC growth rate, and 3) delaying AMR. Thus, theoretically, benzylpenicillin can contribute to combination therapy for MAC-LD via each of the three mechanisms, with drugs such as omadacycline being best partners for future combination studies.^19^

Fourth, we used the γ-based EC_80_ to identify a benzylpenicillin continuous infusion dose of 40 MIU/day for use. The continuous infusion for many months (but not the dose) could be a hardship for some patients, especially when compared to oral medications. This is a potential drawback of using benzylpenicillin for MAC-LD. On the other hand, the drug’s well-known safety profile, which is better than rifamycins and macrolides, would be an advantage.

Finally, γ is the speed of microbial kill, depicted as a trajectory, and is thus a vector, and therefore it can be manipulated using operations (addition, subtraction, multiplication). γ of different exposures can be added to those of a second and third drug in matrices, and factorial design for combination therapy examined in silico. In this case, the different benzylpenicillin γs in the Table can be combined with those of different drugs, such as omadacycline.^19^ This allows for novel in silico-based designs for anti-MAC combination therapy. Thus, we now propose the following programmatic approach: 1) generation of intracellular concentration–response curves in 24-well plates, 2) performing full exposure-surface vs. effect studies in the intracellular HFS-MAC using human lung PK profiles of drugs, 3) performing PK/PD exposure-response analyses based on γ and *m* in tandem with MCE, 4) in silico factorial design-based combinations based on γ, 5) confirmation of combinations vs. SOC in tractable number of HFS-MAC units, and 6) translation to patient SSCC.⁵

There are limitations to our findings. First, the benzylpenicillin half-life of 2.98 hours in HFS-MAC was longer than the 0.5–1.1 h reported in patients and used in our MCE. A recent PK modelling in a patient dataset found a greater than ten-fold difference in benzylpenicillin half-life between individuals.^29^ Second, MIC distribution with many clinical MAC isolates was missing. However, the MCE explored MICs up to 64 mg/L. Third, we tested only one MAC strain in the HFS-MAC. Further work with multiple MAC strains will be needed to generalize as we have used for ceftriaxone elsewhere.⁵ Fourth, in the present study, we found benzylpenicillin accumulated inside infected monocytes, the opposite of what we found in MTB work.^4^ The most likely reason could be that here, we used steady-state concentration measurements after a month of frequent to continuous dosing, while in the former work, sampling was at the beginning of treatment.^4^ Finally, the role of benzylpenicillin in combination therapy is yet to be studied.

In summary, benzylpenicillin achieved >2.0 log_10_ CFU/mL kill against MAC in the HFS-MAC without a β-lactamase inhibitor. The effect of the monotherapy was terminated due to AMR. Exposure-response analysis using γ provided robust and precise estimates of parameters such as EC_50_, H, and E_max_ compared to the same parameters on each sampling day. Finally, we updated our program for developing short-course chemotherapy regimens for MAC lung disease.

## Supplementary Material


