# Supplementary material for: Antibacterial action of penicillin against Mycobacterium avium complex
Source: IJTLD Open. 2024 Aug 1;1(8):362–8. doi: 10.5588/ijtldopen.24.0238 (PMC11308404; doi:10.5588/ijtldopen.24.0238)
Supplement: Supplementary file 1 [file ijtldopen0238_supplementarydata1.docx]

http://dx.doi.org/10.5588/ijtldopen.24.0238

**SUPPLEMENTARY DATA**

**The Antibacterial Action of Penicillin against *Mycobacterium avium-complex***
Devyani Deshpande, Gesham Magombedze, Shashikant Srivastava, Tawanda Gumbo

**SUPPLEMENTARY RESULTS**

**Supplementary Figure S1.** Static penicillin concentration versus microbial effect.

**Supplementary Figure S2.** Pharmacokinetics of benzylpenicillin in the HFS-MAC.

**Supplementary Figure S3.** Biphasic microbial kill in the HFS-MAC by first line drugs.

**Supplementary Figure S4.** Penicillin-resistance emergence in the HFS-MAC using traditional approaches.

**Supplementary Table S1.** Corrected Akaike information criteria score for each PK/PD parameter.

**Supplementary Table S2.** Inhibitory sigmoid model parameter estimates and 95% confidence intervals in the HFS-MAC.

**Supplementary Table S3.** ODE model estimates for azithromycin monotherapy and azithromycin-ethambutol combination.

**SUPPLEMENTARY METHODS**

**Supplementary Figure S1. Static benzylpenicillin concentration versus microbial effect.**


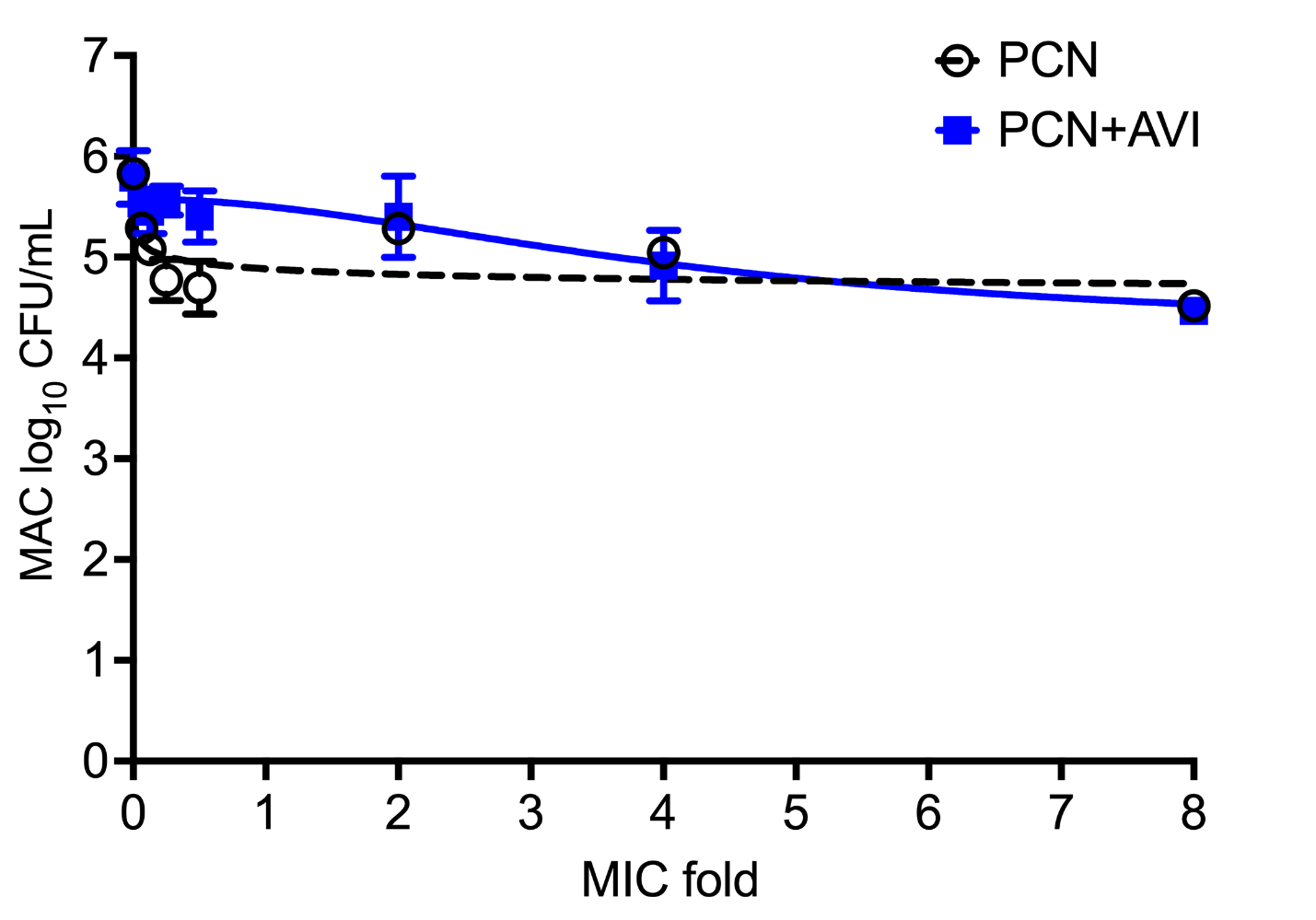


Symbols are mean (n=3), and error bars are standard deviations. Drug concentration is expressed as a ratio of the MIC of 2mg/L. Inhibitory sigmoid E_max_ analyses demonstrated that maximal effect (E_max_) was achieved by 4 times MIC, consistent with time-driven efficacy ^1^. Avibactam did not improve E_max_ or EC_50_ of benzylpenicillin.

**Supplementary Figure S2. Pharmacokinetics of benzylpenicillin in the HFS-MAC.**

**
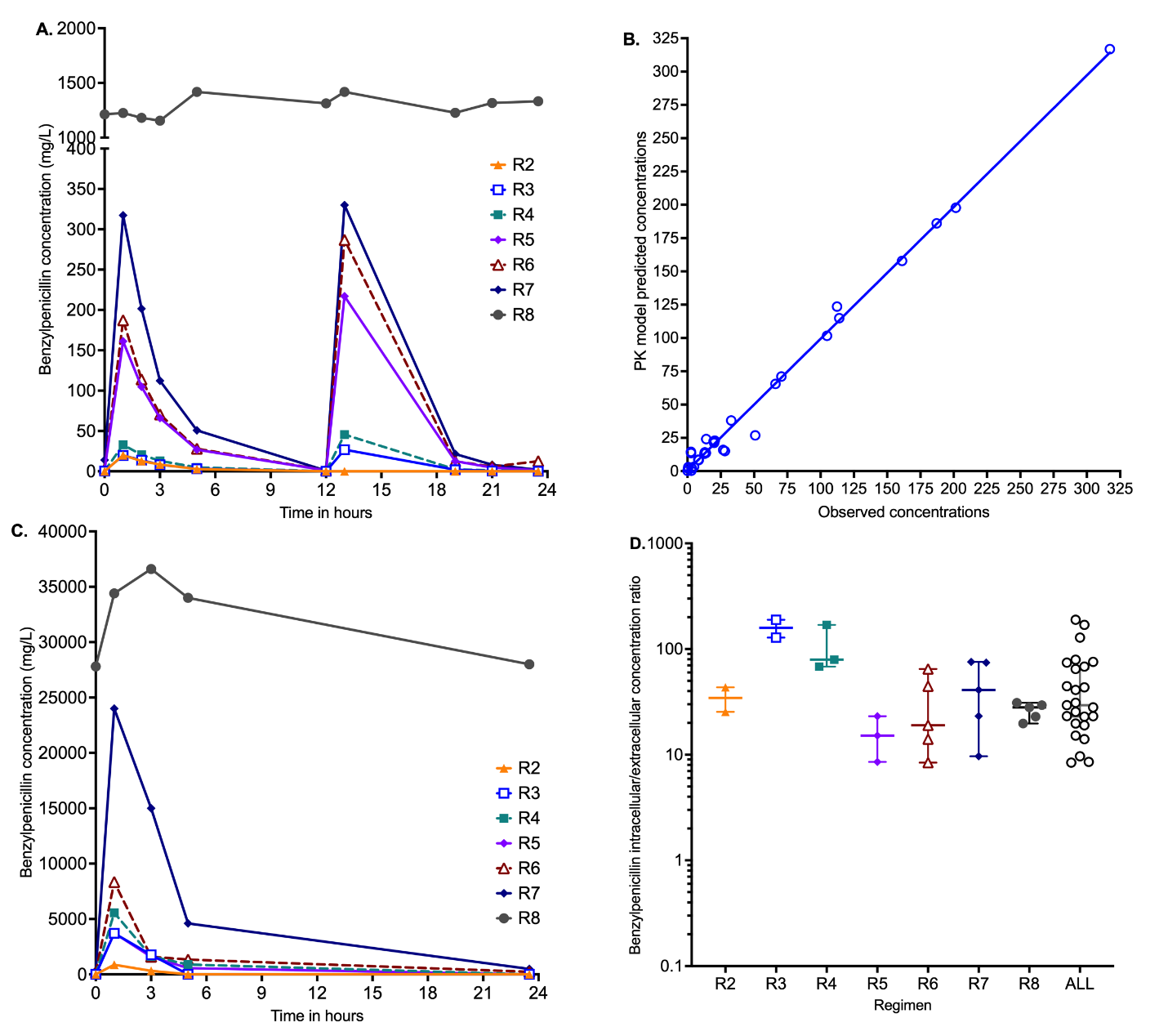
**

**(A)** Drug concentrations were achieved via three different dosing schedules, once a day (R2), twice a day (R3 to R7, and a high continuous infusion (R8). This led to the following exposures as % time concentration persisted above MIC (%T_MIC_), R2 20%, R3 40%, R4 50%, R5 60%, R6 66%, R7 75%, and R8 100%. (**B**) PK model observed versus model predicted concentrations are shown. The linear regression slope was 0.99 (95% confidence interval: 0.95 to 1.03) and r^2^>0.99, indicating minimal bias. (**C**) Intracellular concentrations based on cell volume (L), were multiple fold extracellular ones, and thus Y axis scale is different from panel A. (**D**) Intracellular to extracellular concentration ratio by regimen. Bars are median, and error bars 95% confidence intervals. The ratios were >10 in all regimens.

**Supplementary Figure S3. Biphasic microbial kill in the HFS-MAC by first-line drugs.**

(**A**) The human-equivalent doses of azithromycin were administered in the intracellular HFS-MAC. Azithromycin demonstrated a biphasic effect, with maximal kill below day 0 (stasis) of 0.48 log_10_ CFU/mL, but that at a high dose. (B) Two human-equivalent doses of rifabutin demonstrate that the dose of 300mg did not kill below stasis. (**C**) Ethambutol is shown for only 7 days. Ethambutol 15mg/kg dose did not kill below stasis and was basically parallel to non-treated controls.

**Supplementary Figure S4. Penicillin-resistance emergence in the HFS-MAC using traditional approaches.**


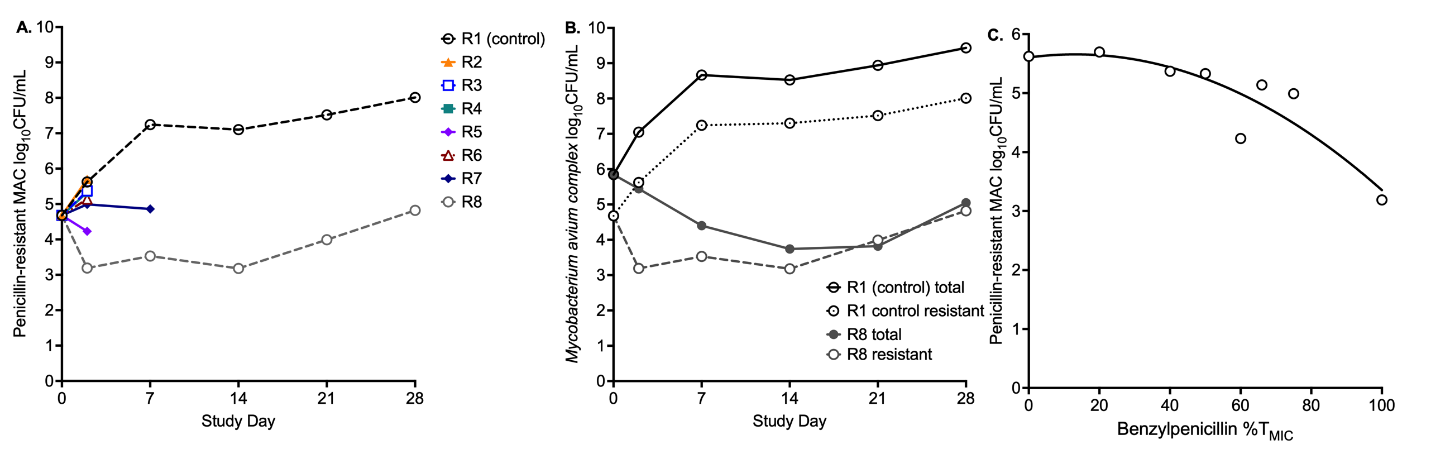


(**A**) Benzylpenicillin-resistant MAC is shown. Benzylpenicillin regimens (R) achieved the following % time concentration persisted above MIC (%T_MIC_) exposures, R1 0%, R2 20%, R3 40%, R4 50%, R5 60%, R6 66%, R7 75%, and R8 100%. For R2-R7, the day 7 drug-resistant population was >5 log_10_ CFU/mL, while that for Days 14-28 was >6.0 log_10_ CFU/mL, based on lowest dilution that had colonies that were too numerous to count on agar. (**B**) The side-by-side comparison of R8 (%T_MIC_=100) total MAC population and the benzylpenicillin-resistant subpopulation shows that why benzylpenicillin killed MAC effectively leading to decline of total bacterial burden from 5.84 log_10_ CFU/mL until 3.47 log_10_ CFU/mL on day 14, the drug-resistant subpopulation stayed flat until day 14 (3.18 log10 CFU/mL, and then started to grow and was 100% of the total population on day 21 onwards. (**C**) Benzylpenicillin exposure versus drug-resistant MAC log_10_ CFU/mL on day 2, modeled using the quadratic function ^2^.

| **Supplementary Table S1. Corrected Akaike information criteria score for each PK/PD parameter.** | | | | | |
| --- | --- | --- | --- | --- | --- |
|  | **Day 2** | **Day 7** | **Day 14** | **Day 21** | **Day 28** |
| C_max_/MIC | 10.74 | 24.50 | 15.37 | 51.50 | 62.90 |
| AUC/MIC | 11.27 | 31.49 | 16.10 | 66.02 | 28.94 |
| %T_MIC_ | **-8.45** | **2.781** | **-2.62** | **5.70** | **23.27** |

Bold Akaike Information Criteria scores indicate PK/PD parameter linked to effect on each sampling day.

| **Supplementary Table S2. Inhibitory sigmoid model parameter estimates and 95% confidence intervals in the HFS-MAC.** | | | | | | | | | |  |
| --- | --- | --- | --- | --- | --- | --- | --- | --- | --- | --- |
|  | **Day 2** |  | **Day 7** |  | **Day 14** |  | **Day 21** | | **Day 28** |  |
|  |  |  |  |  |  |  |  |  |  |  |
| **E_con_ [log_10_ CFU/mL]** | 6.92 | 6.48 to 7.37 | 8.164 | 7.58 to 8.75 | 8.28 | 7.98 to 8.59 | 8.28 | 7.78 to 8.78 | 9.44 | 8.14 to 10.74 |
| **E_max_ [log_10_ CFU/mL]** | 1.60 | Fixed | 4.265 | Fixed | 4.786 | Fixed | 5.12 | Fixed | 4.387 | Fixed |
| **H** | 1.99 | 0.38 to 3.60 | 5.639 | 2.11 to 9.17 | 14.63 | 4.66 to 24.61 | 73.37 | Wide | Imprecise | Imprecise |
| **EC_50_ %T_MIC_** | 46.14 | 12.57 to 79.72 | 65.68 | 55.50 to 75.87 | 82.24 | 75.03 to 89.45 | 97.43 | -6733137 to 6733332 | Imprecise | Imprecise |
| **r^2^** | 0.90 |  | 0.95 |  | 0.98 |  | 0.94 |  | 0.18 |  |

*Imprecise means software output failed to identify a specific parameter value and was reported as unstable.

**Supplementary Table S3. ODE model estimates for azithromycin monotherapy and azithromycin-ethambutol combination.**

| **Parameter** | **Control** | **Azithromycin** | **Azithromycin + Ethambutol** |
| --- | --- | --- | --- |
| $\boldsymbol{r}_{\boldsymbol{w}}$ | 0.69(0.52-0.75) | Fixed | Fixed |
| $\boldsymbol{r}_{\boldsymbol{m}}$ | - | 0.68(0.54-74) | 0.97(0.91-0.98) |
| $\boldsymbol{m}$ | - | 0.004(0.0014-0.017) | 8.32e-5(4.12e-5-1.0e-4) |
| $\boldsymbol{K}_{\boldsymbol{max}}$ | 8.93(8.17-9.76) | 7.87(7.72-8.02) | 8.66(8.04-9.37) |
| $\boldsymbol{\gamma}_{\boldsymbol{s}}$ | - | 0.74(0.65-0.86) | 0.94 (0.82-0.99) |

**SUPPLEMENTARY METHODS**

**Materials**

Benzylpenicillin was obtained from Baylor University Medical Center, Pharmacy (Dallas, TX, USA). Avibactam was synthesized by BOC Sciences (NY, USA). Penicillin G-d7 was purchased from Toronto Research Chemicals (Ontario, Canada). Phorbol myristate acetate (PMA), Roswell Park Memorial Institute (RPMI) 1640 medium, and heat-inactivated fetal bovine serum were purchased from Sigma-Aldrich (St. Louis, MO). Cellulosic hollow-fiber cartridges (catalog number C2008) were purchased from FiberCell (Frederick, MD, USA). THP-1 monocytes were purchased from the ATCC (TIB-202). *Mycobacterium avium* (ATCC#700898) was propagated from stock cultures in all the experiments. In addition to the standard laboratory strain, five clinical MAC strains from our library were utilized in MIC determination experiments.

**MIC and static concentration-response experiments**

Bacteria were grown to log-phase growth in CAMHB, after which turbidity was adjusted to McFarland standard of 0.5. The turbidity-adjusted cultures were diluted 100-fold in CAMHB to achieve a bacterial density of ~10^5^ CFU/mL. Next, 50 µL of benzylpenicillin dissolved in CAMHB and 5%OADC at two times the testing concentration was dispensed into the wells containing 50 µL of MAC to make the final concentrations of 0, 0.0625, 0.125, 0.25, 0.5, 1.0, 2, 4, 8, 16, and 32 mg/L. Non-treated cultures were used as growth controls. The cultures were incubated at 37°C for seven days in sealed plastic bags. On day 7, the 24-well plates were inspected using an inverted mirror, and MIC was defined as the lowest drug concentration that completely inhibited visible microbial growth in the wells.^3^ The experiments were performed twice, with three replicates for each drug concentration.

**Traditional PK/PD modeling for HFS-MAC and static concentrations**

Drug concentrations from HFS-MAC units were co-modeled using ADAPT 5 software (Biomedical Simulations Resources, USC), as a one compartment PK model for both extracellular and intracellular concentrations. The relationship between bacterial burden versus benzylpenicillin concentration or exposure ceftriaxone on a specific sampling day was described by the equation^4^:

Effect (log_10_ cfu/mL) = E_con_-E_max_* EC^H^] / [EC^H^ + EC_50_^H^] #1

where E_con_ is bacterial burden in non-treated controls, E_max_ is maximal effect, EC_50_ is concentration mediating 50% of E_max_, and H is Hill factor.

**Estimation of MAC kill rates and AMR by antibiotics based on ODEs**

A system of two ODEs was used to describe the dynamics of MAC drug susceptible and mutant (resistant) strains, as follows^5,6^

***Logistic growth*** ***Mutation*** ***Kill rate*** ***Bacterial population***  ***Equation***

$\frac{dB_{w}}{dt}=r_{w}B_{w}\left( 1-\frac{B_{w}+B_{m}}{K_{max}} \right) -mB_{w} -\gamma_{s}B_{w}$ Wild type #2

$\frac{dB_{m}}{dt}=r_{m}B_{m}\left( 1-\frac{B_{w}+B_{m}}{K_{max}} \right) +mB_{w}$ Penicillin-resistant #3

The two MAC strains ($\boldsymbol{B}_{\boldsymbol{i}}\boldsymbol{,i=(w,m)}$**,** the wild type and the mutant, respectively) are assumed to follow logistic growth, $\boldsymbol{r}_{\boldsymbol{i}}\boldsymbol{B}_{\boldsymbol{i}}\left( \boldsymbol{1-}\frac{\boldsymbol{B}_{\boldsymbol{w}}\boldsymbol{+}\boldsymbol{B}_{\boldsymbol{m}}}{\boldsymbol{K}_{\boldsymbol{max}}} \right)$ with growth rate represented by $\boldsymbol{r}_{\boldsymbol{i}}$ for each strain and $\boldsymbol{K}_{\boldsymbol{max}}$ is the shared growth limiting capacity. The mutant strain is seeded at rate $\boldsymbol{m}$. Antibiotic treatment induces MAC killing at a rate $\gamma_{s}$ (CFUs per mL/day). If there is no treatment the system is reduced to only one equation with $\gamma_{s}=0$, and $m=0$ without the seeding of the mutant strain.

The model was used to estimate MAC growth, $r_{w}$ and $K_{max}$ for the wild type using the HFS-MAC non-treated controls (regimen R1). Second, the drug kill rates, $\gamma_{s},$ the mutation rate and growth rate of the mutant, for each exposure are estimated by fitting the model to the experimental data sets R2 to R8 of benzylpenicillin treatment, the azithromycin treatment, and the combination of azithromycin and ethambutol. A gaussian likelihood with Markov Chain Monte Carlo (MCMC) algorithm was applied as explained in Magombedze et al.,^7^ using the R FME package. Parameter posteriors distributions were drawn from 25000 MCMC samples and 95% credible intervals were used to determine the uncertainty in the parameter estimates using 2.5-97.5 quantiles.

**Monte Carlo experiments (MCE) for dose finding.**

The HFS-MAC used different dosing schedules: the intent of the HFS-MAC work was to (i) identify the PK/PD linked effect based on different dosing schedules (bolus once daily or twice daily versus continuous infusion), and (ii) the EC_80_. The HFS-MAC is a deterministic system in which clearance and volume of distribution have minimal variability. This information cannot be attained by examining static concentrations in 24-well plates or test tubes, because drug elimination rates are important to an antibiotics’ performance. The intent of the MCE was not to mimic or mirror HFS-MAC exposures but rather to identify the best human dose that would achieve or exceed the EC_80_ in lungs of patients that had been identified in patients, given population pharmacokinetic parameters (estimates and between-patient variability) of benzylpenicillin encountered in patients and the PK/PD parameter linked to efficacy.

The target exposure was then used in Monte Carlo experiments, to identify the target attainment probability in lungs of 10,000 patients treated with various doses of benzylpenicillin as a continuous infusion. We used the following assumptions. First, as noted in the past the benzyl ester of penicillin achieves high penetration ratio in the lung, especially in pneumonia, to a lung-to-serum ratio of >2.^8-12^ Second, while penicillin is 50% protein bound, the mean protein ELF/plasma ratios even in severe acute respiratory distress syndrome vary from 0.13-0.25, and thus penicillin protein binding in ELF is expected to be negligible, and the measured total antibiotic concentrations in ELF are considered to reflect free (unbound) fractions ^13-15^. We entered the following PK parameter estimates in subroutine PRIOR of ADAPT: a clearance of 21 L/h (interindividual variability [IIV] =33.1%) and a volume of 28.9 L (IIV=32.4%).^16^ These parameter values were generated using NONMEM, which a parametric method that assumes that the population PK parameter distribution is Gaussian, with final structural model that provides separate estimates of a fixed effect and a random effect for each PK parameter, and provides the standard deviation (SD; ω) or the variance (ω2) of random effects. ADAPT 5 version of ADAPT, used for MCE described here, has expanded capabilities that include parametric population PK modeling. For %T_MIC_ linked drugs with a short half-life, the longer the infusion time the more likely to achieve the %T_MIC_ EC_80_ exposure, and continuous infusions are preferred.^17^ Therefore, we decided *a priori* to examine a continuous infusion schedule in our MCE of virtual patients. Continuous infusion rates (mg/h) used in the clinic and dose were entered and specified in model input information (Input Event Information) in the ADAPT .dat file. Doses of 5, 10, 20, 24 and 40 MIU per day were administered as a continuous infusion, and the 24h concentration-time profiles generated for the lung for each dose, in 10,000 subjects. PK/PD target was the EC_80_ %T_MIC_ in lungs of subjects.

**REFERENCES**

1. Ambrose PG, Bhavnani SM, Rubino CM, et al. Pharmacokinetics-pharmacodynamics of antimicrobial therapy: it's not just for mice anymore. *Clin Infect Dis.* 2007;44(1):79-86.

2. Schmalstieg AM, Srivastava S, Belkaya S, et al. The antibiotic resistance arrow of time: efflux pump induction is a general first step in the evolution of mycobacterial drug resistance. *Antimicrob Agents Chemother.* 2012;56(9):4806-4815.

3. CLSI. *Susceptibility testing of mycobacteria, nocardiae, and other aerobic actinomycetes, 3rd Edition.* Wayne, PA: Clinical and Laboratory Standards Institute;2018.

4. Deshpande D, Srivastava S, Gumbo T. A programme to create short-course chemotherapy for pulmonary *Mycobacterium avium* disease based on pharmacokinetics/pharmacodynamics and mathematical forecasting. *J Antimicrob Chemother.* 2017;72(suppl_2):i54-i60.

5. Deshpande D, Magombedze G, Boorgula GD, Chapagain M, Srivastava S, Gumbo T. Ceftriaxone efficacy for Mycobacterium avium complex lung disease in the hollow fiber and translation to sustained sputum culture conversion in patients. *J Infect Dis.* 2023.

6. Chapagain M, Pasipanodya JG, Athale S, et al. Omadacycline efficacy in the hollow fibre system model of pulmonary Mycobacterium avium complex and potency at clinically attainable doses. *J Antimicrob Chemother.* 2022;77(6):1694-1705.

7. Magombedze G, J P, Srivastava S, Deshpande D, McIlleron H, Gumbo T. Transformation morphisms and time to extinction analysis that map therapy duration from pre-clinical models to patients with tuberculosis: translating from apples to oranges. *Clin Infect Dis.* 2018;67(Suppl 3):S349-S358.

8. Zeitlinger MA, Derendorf H, Mouton JW, et al. Protein binding: do we ever learn? *Antimicrobial Agents and Chemotherapy.* 2011;55(7):3067-3074.

9. Craig WA, Kunin CM. Significance of serum protein and tissue binding of antimicrobial agents. *Annu Rev Med.* 1976;27:287-300.

10. Ungar J, Muggleton PW. Accumulation of diethylaminoethanol ester of penicillin in inflamed lung tissue. *Br Med J.* 1952;1(4770):1211-1213.

11. Heathcote AG, Nassau E. Concentration of penicillin in the lungs. Effects of two penicillin esters in chronic pulmonary infections. *Lancet.* 1951;1(6667):1255-1257.

12. Jensen KA, Dragsted PJ, Moller P, Klaer I. Investigations of penicillin preparations and dosage schedules. *Ugeskr Laeger.* 1950;112(30):1043-1046.

13. Grigg J, Kleinert S, Woods RL, et al. Alveolar epithelial lining fluid cellularity, protein and endothelin-1 in children with congenital heart disease. *Eur Respir J.* 1996;9(7):1381-1388.

14. Holter JF, Weiland JE, Pacht ER, Gadek JE, Davis WB. Protein permeability in the adult respiratory distress syndrome. Loss of size selectivity of the alveolar epithelium. *J Clin Invest.* 1986;78(6):1513-1522.

15. Kiem S, Schentag JJ. Interpretation of antibiotic concentration ratios measured in epithelial lining fluid. *Antimicrobial Agents and Chemotherapy.* 2008;52(1):24-36.

16. Komatsu T, Inomata T, Watanabe I, et al. Population pharmacokinetic analysis and dosing regimen optimization of penicillin G in patients with infective endocarditis. *J Pharm Health Care Sci.* 2016;2:9.

17. Craig WA, Ebert SC. Continuous infusion of beta-lactam antibiotics. *Antimicrob Agents Chemother.* 1992;36(12):2577-2583.
